# Recent trends in pharmaceutical freeze-drying and control strategies observed in human drug applications and manufacturing inspections

**DOI:** 10.1186/s41120-025-00132-4

**Published:** 2025-12-01

**Authors:** Steve Y. Rhieu, Maxwell Korang-Yeboah, David D. Anderson, John Arigo, Thomas O’Connor, Rakhi Shah

**Affiliations:** 1 Office of Pharmaceutical Manufacturing Assessment, Office of Pharmaceutical Quality, Center for Drug Evaluation and Research, Food and Drug Administration, 10903 New Hampshire Avenue, Silver Spring, MD 20993, USA; 2 Office of Pharmaceutical Quality Research, Office of Pharmaceutical Quality, Center for Drug Evaluation and Research, Food and Drug Administration, 10903 New Hampshire Avenue, Silver Spring, MD 20993, USA

**Keywords:** Freeze-drying, Lyophilization, Manufacturing controls, Continuous manufacturing, Emerging technologies

## Abstract

Freeze-drying has been a workhorse for the pharmaceutical industry to enable long-term storage and long-distance transport of drug products that are unstable in liquid or frozen form. The importance of freeze-drying was particularly evident in the development and storage of life-saving medicines such as mRNA-based vaccines during the COVID-19 pandemic. In response to growing demand for improved manufacturing efficiency, emerging freeze-drying technologies spurred by the U.S. Food and Drug Administration (FDA) Pharmaceutical Quality for the 21st Century Initiative have been introduced with the aim to modernize the pharmaceutical manufacturing sector. Here, we provide regulatory considerations for emerging technologies in pharmaceutical freeze-drying from a manufacturing perspective. We also present an analysis of current trends in freeze-drying and in-process controls submitted in human drug applications—24 New Drug Applications, 118 Abbreviated New Drug Applications, and 20 Biologics License Applications assessed between 2020 and 2023—seeking FDA approval. Additionally, we analyzed the Form FDA-483 Inspection Observations educed from 201 inspections conducted at manufacturing facilities performing freeze-dried parenteral operations between 2015 and 2019, to identify common compliance challenges and provide regulatory insights for the pharmaceutical industry.

## Introduction

**Fig. 1:**
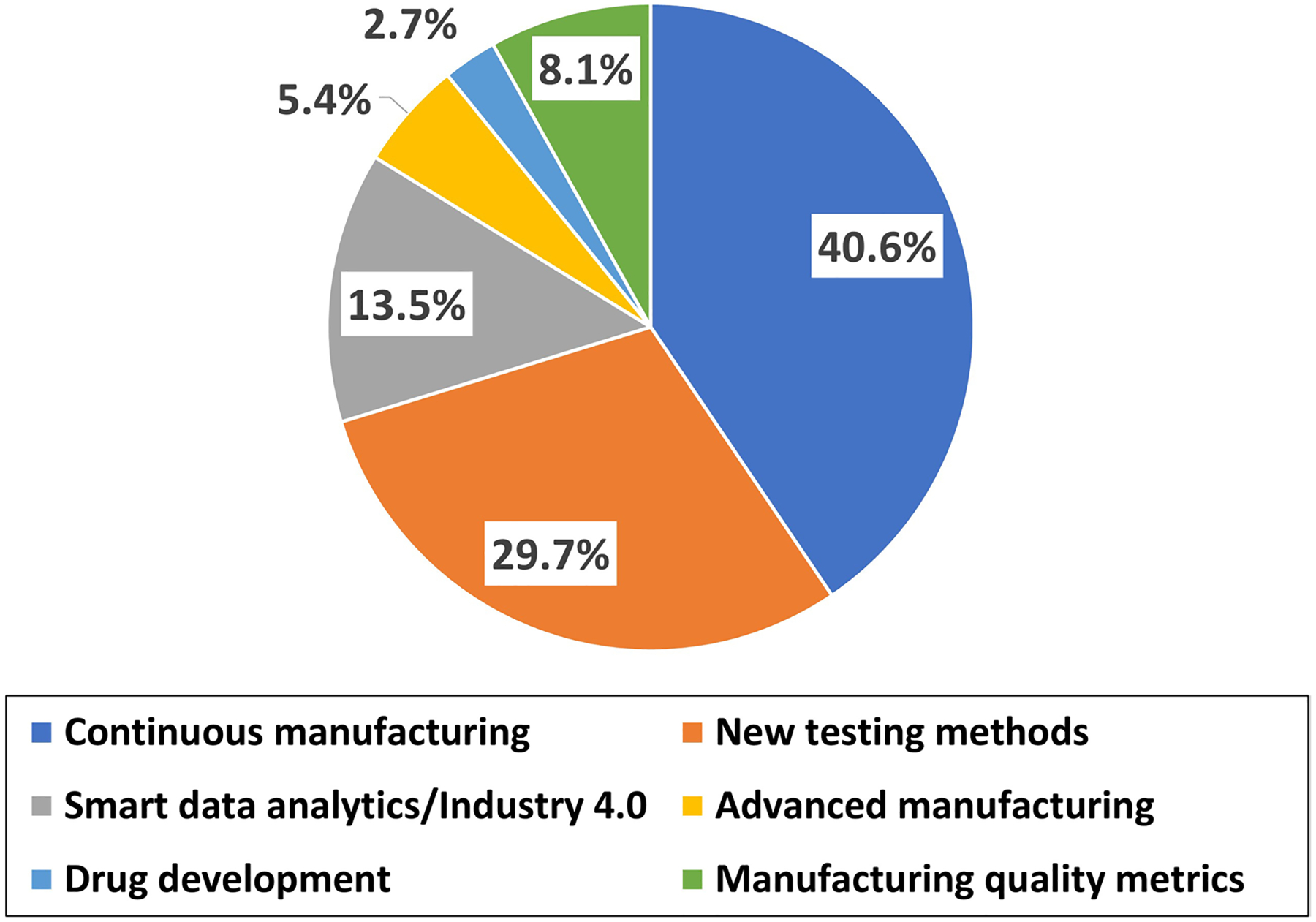
Extramural research projects funded by CDER’s Office of Pharmaceutical Quality from 2017 to 2023 (*n* = 37). Three projects focusing on topics of continuous manufacturing, new testing methods, and advanced manufacturing are related to freeze-drying

Freeze-drying, also known as lyophilization or cryodes-iccation, transforms a drug formulation from a liquid to a stable solid by removing water or other solvents. As a result, freeze-dried drugs can have a longer shelf life and improved stability. Owing to such benefits, freeze-drying has widely been applied to heat- and moisture-sensitive drug formulations such as antibiotics, peptides, proteins and vaccines. In recent years, the surge in demand for freeze-drying has been driven in part by the COVID-19 pandemic response and recovery in parallel with future pandemic preparedness. To this end, the U.S. Food and Drug Administration (FDA) has long championed the adoption of emerging technologies that would lead to agile and flexible manufacturing processes to produce high-quality drugs (FDA 2004a). For example, the Center for Drug Evaluation and Research (CDER) of the FDA created the Emerging Technology Program (ETP) to facilitate discussions on technical and regulatory aspects of innovative approaches to manufacturing (e.g., controlled ice nucleation for freeze-drying) between the agency and industry representatives prior to filing a regulatory submission. In addition, extramural research projects designed to modernize manufacturing approaches and invigorate public health preparedness and response have been funded by CDER’s Office of Pharmaceutical Quality. Of the 37 projects funded from 2017 to 2023, three research projects (8%) pertain to freeze-drying focusing on topics of continuous manufacturing, new testing methods, and advanced manufacturing (Fig. 1). In continuation with the FDA’s efforts to help the industry adopt emerging manufacturing technologies, this report provides considerations for regulatory and technical challenges associated with promising approaches to freeze-drying. Provided is also a trend analysis of manufacturing process controls for freeze-drying submitted in regulatory submissions seeking FDA approval as well as drug product manufacturing inspections.

## Methods

We examined a total of 162 regulatory submissions comprising 24 New Drug Applications (NDAs), 118 Abbreviated New Drug Applications (ANDAs), and 20 Biologics License Applications (BLAs) assessed between 2020 and 2023. The analysis focused exclusively on lyophilized drug products submitted to CDER for evaluation and approval. Supporting information used for the analysis was obtained from the FDA’s internal database.

## Results

### Manufacturing control trends in regulatory submissions

**Fig. 2:**
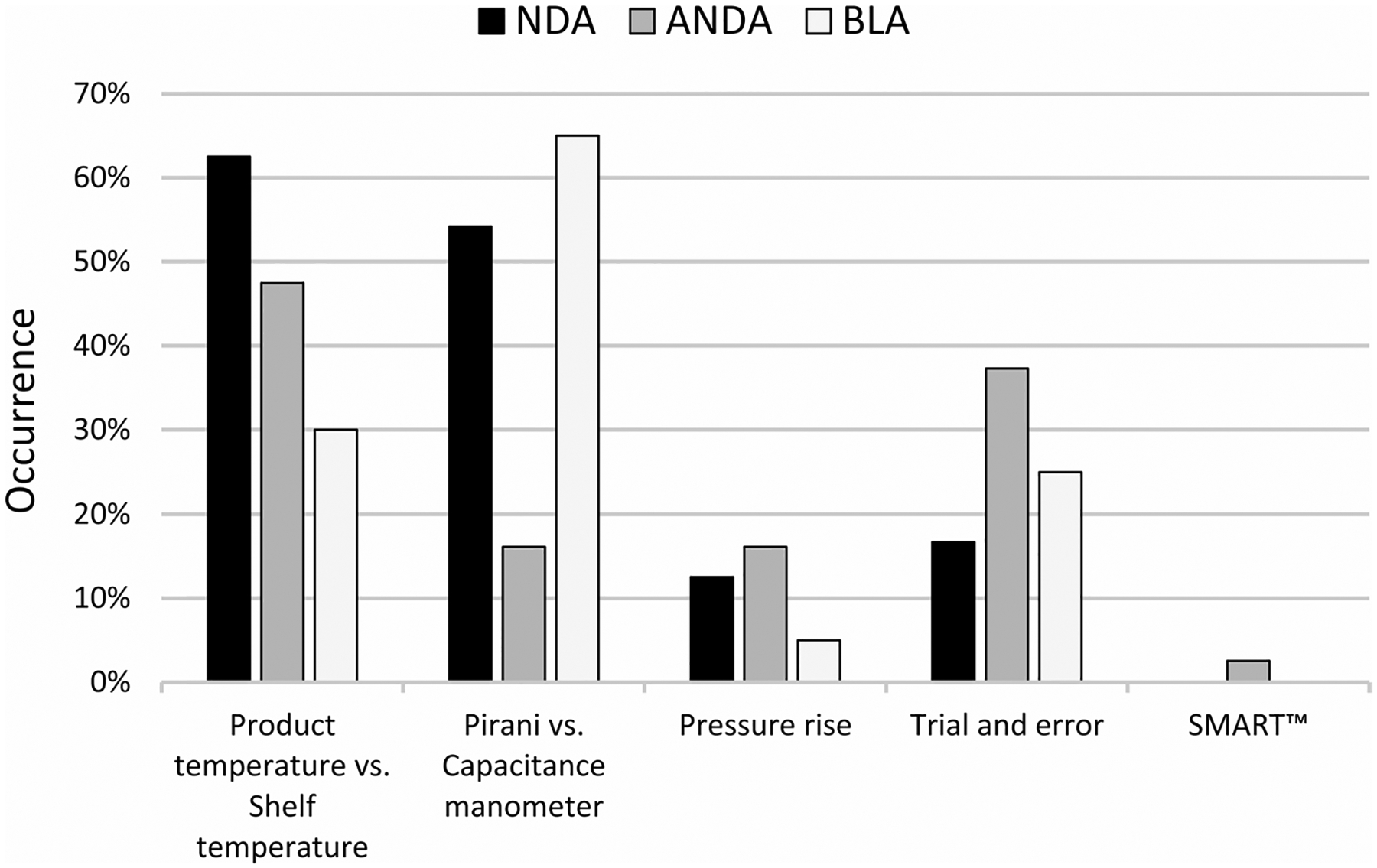
Methods used to determine the endpoint of drying observed from 24 New Drug Applications (NDAs), 118 Abbreviated New Drug Applications (ANDAs), and 20 Biologics License Applications (BLAs) examined

The endpoint of the sublimation process is an important control point in the freeze-drying process and is typically monitored through the product temperature and pressure changes during the lyophilization cycle. Figure 2 shows different methods used to monitor the progress of primary and secondary drying during lyophilization cycle development for 24 NDAs, 118 ANDAs, and 20 BLAs examined. Direct measurement of product temperature approaching shelf temperature was the most common endpoint determination method, used to avoid product melt back or collapse. This approach was employed by 62.5% of NDAs and 47.5% of ANDAs. Other process analytical technology (PAT) tools were also used, including pressure rise test and comparative pressure measurement via Pirani gauge and capacitance manometer—the latter being the most popular method among BLAs. It is worth noting that SMART^™^ (Tang et al. 2005; Gieseler et al. 2007a), a manometric temperature measurement-based model designed to maintain the product temperature below its collapse temperature during drying, was used for relatively few cases, representing only 2.54% of ANDAs. A trial-and-error approach to lyophilization cycle development was also frequently observed across all application types, where for a given collapse temperature of the formulation, experiments were performed with various shelf temperature and chamber pressure values until the acceptable output was obtained. This approach was used by 16.7% of NDAs, 37.3% of ANDAs, and 25.0% of BLAs.

Regarding scaling up of the freeze-drying process, both NDAs (33.3%) and ANDAs (22.9%) pursued commercial manufacturing with a scale-up factor of 1.5 to 10 in accordance with the FDA’s guidance on batch size (FDA 2014). Notably, these applications maintained the same equipment as well as process parameters used for the manufacture of exhibit batches. For BLAs, it is expected that process performance qualification (PPQ) studies at commercial scale be performed prior to submission and that PPQ data be provided in the application for evaluation as per 21 CFR 601.20(b) and other applicable regulations. Drug applicants are expected to demonstrate that the process is capable of reproducible commercial manufacturing by leveraging the knowledge gained from development and exhibit batch manufacturing. Lack of data or justification to support scale-up strategies may lead to the issuance of deficiencies (Rhieu et al. 2020). Despite the availability of various PAT tools, their use in commercial manufacturing was infrequent with only 4.17% of NDAs and 0.85% of ANDAs incorporating PAT tools in manufacturing processes. Examples of available tools include tunable diode laser absorption spectroscopy (Gieseler et al. 2007b), near-infrared spectroscopy (De Beer et al. 2009), and wireless product temperature sensors (Schneid and Gieseler 2008; Juckers et al. 2023). Among PAT tools proposed in applications for commercial manufacturing, pressure rise tests were the most common (13% of NDAs; 16% of ANDAs; and 5% of BLAs), while SMART^™^ was proposed in 3% of ANDAs. These tools were intended for the timely analysis of drying endpoints and control loops to adjust the processing conditions, respectively.

### Trends in form FDA-483 inspection observations

Over the last 30 years, only a handful of documents have been published that outline the pharmaceutical industries thinking related to good manufacturing practices (GMPs) on lyophilized parenterals. In 1993, the FDA published an inspection technical guide on how to conduct inspections of lyophilized parenterals (FDA 1993). This guide provided a broad overview of the critical concepts of manufacturing lyophilized parenterals from both a chemical and microbial control viewpoint across the filling operation, the transport of filled vials to the lyophilizer, and execution of the lyophilization cycle. The importance of formulation design, product specific lyo cycle development, lyophilizer sterilization, and finished product testing including visual inspection were also discussed. Later in 2004, the FDA published a guidance on the current GMPs for manufacturing of sterile drug products and more recently in 2022 the European Union GMP Annex 1 on the manufacturing of sterile medicinal products was released both of which include specific considerations for the manufacturing of lyophilized parenterals (FDA 2004b; EU 2022). Together these documents outline the fundamental aspects of manufacturing lyophilized parenterals in a current GMP compliant environment.

**Fig. 3:**
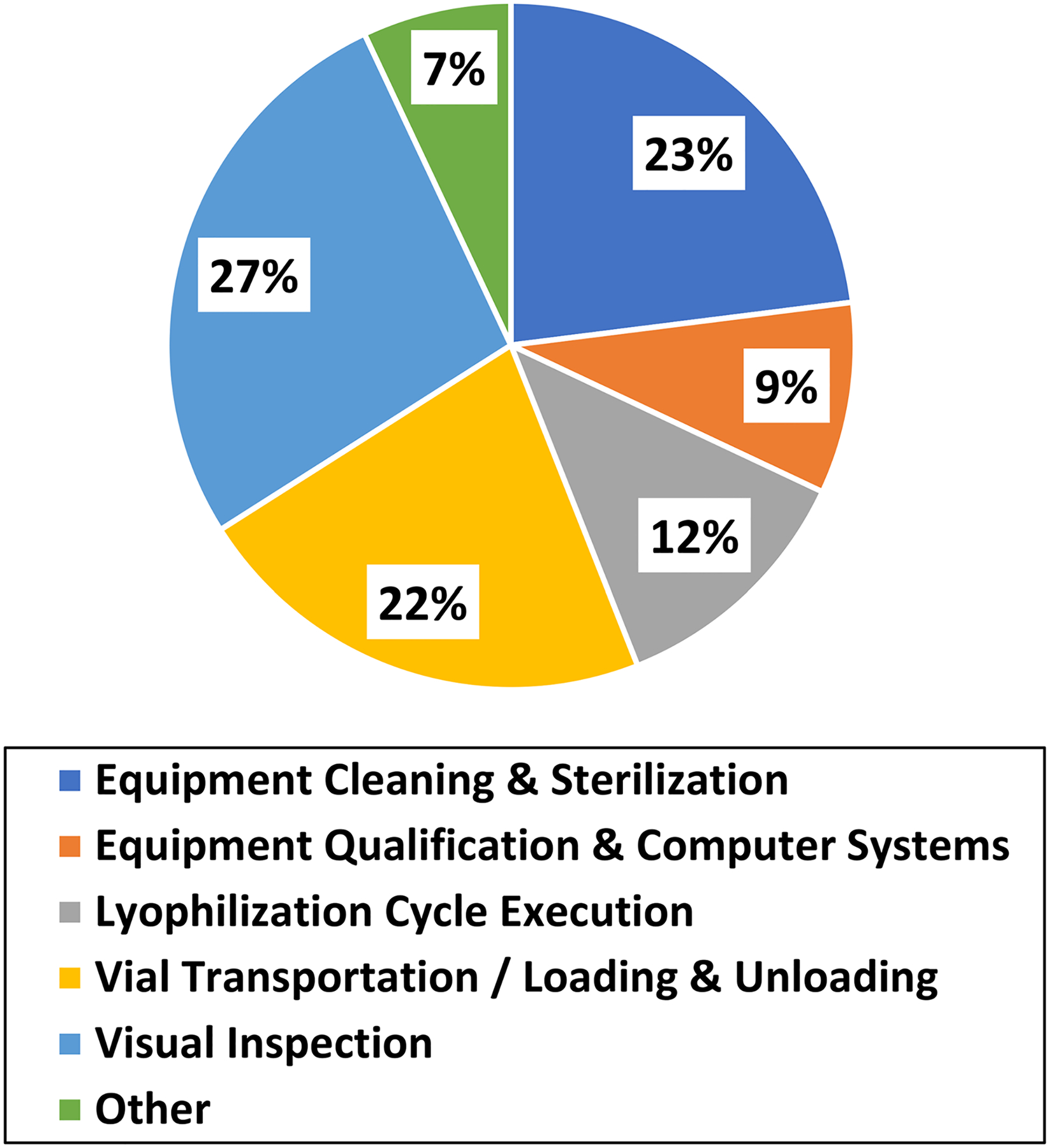
Recent trends in 483 observations related to lyophilized drug products. Commonly cited objectionable conditions for visual inspection included: (1) inadequate defect libraries for training, (2) product complaints, AQL failures, failures observed in sample retains and stability samples indicative of inadequate training, (3) USP recommendations (inspection time per vial, operator fatigue, light intensity, black/white inspection background) not followed. For equipment cleaning, sterilization & aseptic practices: (1) lack of integrity testing of sterilizing filters for backfill gas (e.g., nitrogen), operators self-sanitizing immediately before personnel monitoring, inadequate placement of non-viable probes and settle plates. For transportation and loading of partially stoppered vials from filling into the lyophilizer: (1) first air breached unloading trays from HEPA cart or loading trays into the lyophilizer, (2) inadequate or absence of smoke studies. For execution of the lyophilization cycle: (1) critical process limits not defined, (2) lack of review of process data, (3) absence of product specific development data to support lyophilization cycle, (4) inadequate investigations into process deviations. For equipment and computer system qualification: (1) user access permissions not restricted by role and (2) data retrieval issues. For other: (1) lack of trending of complaints and (2) insufficient investigations or CAPAs related to lyophilization processes

To assess the current compliance status of the pharmaceutical industry related to lyophilized parenteral manufacturing, we reviewed 201 FDA establishment inspection reports (EIRs) of inspections conducted at drug product manufacturing facilities performing lyophilized parenteral operations between 2015 and 2019. Of the EIRs reviewed, 104 (52%) included an average of 1.8 objectionable conditions directly tied to the lyophilization unit operation and cited as 483 observations. When categorized by manufacturing activity (Fig. 3), there are three main areas that account for approximately 75% of the reported 483 observations including: (1) the visual inspection program; (2) equipment cleaning, sterilization, and aseptic techniques; and (3) transfer of partially stoppered vials from the filling operation and loading into the lyophilization chamber. Observations related to the execution of the lyophilization cycle (12%) or qualification of the lyophilization equipment and computer system (9%) were less frequently cited. Overall, the visual inspection of lyophilized parenterals was the most common source of 483 observations. For firms seeking to improve their compliance in this area, the USP chapters <790> and <1790> as well as a recently published paper on acceptable lyophilized cake appearance may be useful resources (Patel et al. 2017).

## Discussion

### Sterilization of the lyophilizer and aseptic control of the lyophilization process

The lyophilization machinery is part of the aseptic filling line and as such it needs to be considered when designing the process validation studies to ensure sterility assurance. Containers enter the lyophilizer partially stoppered and are not fully sealed so there is risk for microbial contamination if the manufacturing process is not well controlled. Sterilization-in-place (SIP) is the most commonly seen method for sterilization of the lyophilizer system. In designing the SIP validation, temperature and time should be carefully considered as well as the inclusion of biological indicators that are incubated for seven days after SIP exposure as per USP <55> and ISO 11138. Validation reports typically include data from lyophilization performed with parameters that are either identical or worst case compared to commercial production.

When designing the overall aseptic control program for a lyophilizer manufacturers should consider the environment the machine is in and the environment the partially stoppered vials travel within to get to the lyophilizer. The lyophilizer should provide for an ISO 5/Grade A environment during lyophilization as well as during the transport and loading of the lyophilizer (FDA 2004b). An evaluation of how containers are transported and loaded into the lyophilizer and the environment they experience during the transport and loading steps is generally needed with an appropriate justification of these steps and the aseptic controls used. In addition, suitable risk assessment would include all steps of the process. For example, if a mobile cart with laminar air flow is utilized, then this needs to be included in the risk assessment provided to the Agency. A common deficiency observed in our analysis was lack of confirmation that the same type of lyophilizer used in the aseptic process simulation studies was also proposed for commercial production. The transport of partially stoppered vials, loading of the lyophilizer, drawing a partial vacuum and breaking the vacuum with sterile air, as well as unloading the lyophilizer should be included in the aseptic process simulation, which is typically why the same type of machinery is recommended to be used for commercial production (PDA 2011). Any holding periods between the end of filling and the start of lyophilization should also be simulated as closely as possible as the partially stoppered vials will not necessarily be held in a sterile environment. Lastly, if the lyophilizer is manually loaded, how the lyophilizer trays and loading equipment are sterilized should be explained.

### Emerging technology trends in freeze-drying

Since the early 2000s, the FDA has actively promoted for the adoption of emerging technologies in pharmaceutical manufacturing through initiatives such as the Pharmaceutical Quality for the 21st Century program. Specially for freeze-drying applications, the following emerging technologies represent key enablers that have been introduced to promote pharmaceutical innovation and modernization, though this list is not exhaustive.

### Controlled ice nucleation

It is known that the stochastic nature of ice formation and higher degree of supercooling during the conventional freezing lead to higher inter- and intra-batch heterogeneity in drying behaviors and product quality attributes (Searles et al. 2001). Further, the conventional freezing process results in smaller dendritic ice structures, smaller air-void in the dry layer, a higher resistance to sublimation and prolonged primary drying process (Assegehegn et al. 2019; Searles et al. 2001). To overcome these challenges, various techniques have been developed for controlling ice nucleation during the freezing step of lyophilization. The controlled ice nucleation (CIN) techniques are aimed at inducing concurrent ice nucleation in all vials at a consistent time and ice nucleation temperature, decreasing heterogeneity in nucleation temperatures and product’s quality attributes. By reducing the degree of supercooling, CIN may significantly reduce primary drying time and improve product quality (Korang-Yeboah et al. 2023; Ma et al. 2025). Several CIN techniques have been studied at various scales and stages of pharmaceutical manufacturing. These techniques include, but are not limited to, pressurization depressurization, ice fog, thermo-mechanical shocks, and vacuum induced techniques. Currently, rapid pressurization-depressurization (i.e., ControLyo^®^) and ice fog (i.e., VERISEQ^®^) have been built into certain commercial lyophilizers (Assegehegn et al. 2019). Our analysis indicates that the use of CIN has been approved for one NDA and one BLA since 2020 and that three applications on the adoption of CIN have been evaluated through CDER’s ETP.

### Process analytical technology

The Office of Pharmaceutical Quality within CDER defines pharmaceutical quality as assuring every dose is safe, effective, and free of contamination and defects. As a practical matter, only a certain number of end products can be tested so overall product quality assurance built into the lyophilization process is important for assurance that all the drug products produced from this step are of suitable quality. This approach is in alignment with the core principle of the Quality by Design (QbD) paradigm which calls for a systematic, scientific, and risk-based approach to product and process design and manufacturing that provides process understanding and consistently yields product meeting the target product quality profile. One way of achieving the desired QbD targets is the use of integration of data from PAT tools for real time process monitoring and control. The implementation of PAT further enhances the understanding of the relationship between product and process parameters and their interaction on critical quality attributes of the product. There has been an increasing adoption of PAT in the pharmaceutical industry since the proposal of the QbD-PAT initiative (Alam et al. 2021; Kim et al. 2021; Bekaert et al. 2022; Krogmeier et al. 2022). However, the adoption of PAT for lyophilization process monitoring and control of lyophilization at commercial scale is still limited, specifically for legacy products and generic pharmaceutical products (Rhieu et al. 2020; Stamato et al. 2025). Over the last two decades the value addition of integrating PAT tools at different stages and scales of lyophilization has been demonstrated with a variety of techniques including, but not limited to, comparative pressure monitoring, manometric temperature monitoring, tunable diode laser absorption spectrometry, dew-point analysis, residual gas analysis, wireless temperature monitoring, heat flux sensors, gravimetric or weight loss technique (Wang and Tang 2023). Here we focus on a few of the PAT tools with lower barriers to adoption for real time monitoring and controlling of commercial scale lyophilization processes.

### Comparative pressure or differential pressure monitoring

The comparative or differential pressure monitoring technique takes advantage of the difference in mechanism of pressure measurement by the capacitance pressure gauge and Pirani gauge for determining the end of primary drying. The capacitance manometer is an absolute pressure measurement technique that is independent of the gas composition in the freeze dryer during primary drying. Pirani gauges, on the other hand, are thermal conductivity gauges, thus Pirani pressure reading is dependent on the gas composition in the freeze dryer chamber. As the gas composition changes from predominantly water vapor to nitrogen as the process transitions from primary drying to secondary drying, the difference in pressure measurement of the Pirani gauge and capacitance manometer may be used for process monitoring and determining the end of primary drying (Nail et al. 2017; Korang-Yeboah et al. 2018). Furthermore, the differential pressure between the chamber and condenser measured by capacitance manometers may have added benefit in leak detection, troubleshooting and early detection of equipment failure, and potentially monitoring the rate of sublimation during commercial manufacturing (Authelin et al. 2024).

### Tunable diode laser absorption spectroscopy

Tunable Diode Laser Absorption Spectroscopy (TDLAS) is a near-infrared absorption spectroscopy technique used for real time measurement of the water vapor mass flow rate from the drying chamber to the condenser of the freeze-dryer. The TDLAS is compatible with both laboratory- and commercial-scale dryers. The TDLAS unit is installed in the spool separating the chamber and condenser and thus incompatible with freeze dryers with no optical spool. The TDLAS allows direct measurement of the maximum sublimation rate of commercial-scale freeze-dryers during operational qualification, supports process development, cycle optimization, transfer and scale up. Furthermore, a combination of the TDLAS measurement with simple steady state models enables real time measurement of batch average product temperature, product resistance to drying, determination of the end of primary and secondary drying, and potentially prediction of the batch average moisture content (Gieseler et al. 2007a; Korang-Yeboah et al. 2023; Ganguly et al. 2023; Jameel et al. 2023). In a recent study, the TDLAS was employed for monitoring and control heterogeneity in drying kinetics of a freeze-drying process (Yu et al. 2024). The TDLAS may ease the challenges with equipment and process characterization at commercial scale, including, but not limited to, the determination of choke flow and the maximum sublimation rates commercial dryers can handle without loss of process control.

### Wireless temperature monitoring systems

The product temperature is the most important product parameter during freeze-drying. However, product temperature monitoring at commercial scale is limited due to the practical limitations of conventional wired thermocouples and resistance temperature detector sensors. Traditional wired thermocouples are challenging to implement at commercial scale. First, wired temperature measurement tools are generally incompatible with automatic loading systems and require manual insertion of probes in multiple products. This manual step increases the risk of product contamination. Furthermore, only a limited number of products can be monitored by wired temperature tools as products farther away from the front rows of the freeze dryer are inaccessible to manual operators (Nail et al. 2017). Challenges with wire management and potential tipping of product vials are additional limitations of traditional wired thermocouples. These challenges have necessitated the development of wireless temperature monitoring systems compatible with automated filling lines and are machine operable. One of such technologies is the temperature remote interrogation system or TEMPRIS. At the heart of TEMPRIS system is a quartz sensing element whose oscillation at a characteristic frequency is dependent on temperature. These battery-free sensors are powered by excitation of the passive transponder by means of an amplitude-modulated electromagnetic signal in the internationally available 2.4-GHz band. TEMPRIS has been shown to be as accurate as traditional thermocouple for product temperature measurement and determination of the end of primary drying when placed at the bottom center of the vial (Schneid and Gieseler 2008; Patel et al. 2009). To our knowledge, TEMPRIS is currently being used in commercial-scale manufacturing for the European market (Searles and Hufnagel 2023).

The implementation of emerging technologies such as CIN and PAT may help increase the assurance of product quality and reduce the risk of product shortages. However, the adoption of such technologies may present additional risks to product quality and process performance that needs to be identified and controlled (ICH Q9). Therefore, there is a need for process and technology understanding, identification of potential failure modes, risk to product quality and process efficiency, and implementation of adequate risk mitigation strategies. Per a recent publication by the FDA (Korang-Yeboah et al. 2023), for the added benefits of CIN to be attained, there is a need for a robust process design that considers the impact of the primary and secondary drying process parameters on process efficiency and product quality. For example, a uniform and rapid distribution of ice fog throughout the chamber during the ice nucleation step may be necessary for ice nucleation to occur within the desired set time (Pisano 2019; Ganguly 2024). Process characterization studies at commercial scale may be useful in understanding ice flow pattern and distribution during induction of ice nucleation. Similarly for a CIN process using the pressurization-depressurization technique, the rate of depressurization has been reported to impact the ability to homogeneously induce ice nucleation in all product vials (Luoma et al. 2019; Strongrich et al. 2021). Furthermore, an internal FDA study revealed the shelf ramp rate post ice nucleation and addition of extra hold time may impact the ice crystal size, product resistance, sublimation rate, primary drying time, and product attributes (Luoma et al. 2019; Korang-Yeboah et al. 2019; Fang et al. 2020). These examples underscore the importance of process understanding prior to implementation of CIN. Moreover, the product vials are partially stoppered during freeze-drying. This increases the risk of contamination when any tool or material is placed in the freeze-drying chamber during the process. The risk may be higher for PAT tools that come in direct contact with the product, or CIN techniques that induces ice nucleation via introduction of an external material into the freeze-dryer. For this reason, it may be necessary to evaluate the impact of CIN processes or PAT implementation on product sterility.

### Process modeling

Process models are increasingly employed to enhance and expedite process design, scale-up, site transfer, process monitoring, and control. In addition, it has been demonstrated that prediction of critical process parameters using process modeling can be a part of an enhanced QbD approach for lyophilized products (Mockus et al. 2011). Primary drying, being the most time-consuming and energy-intensive phase of lyophilization, is the principal focus for process optimization (Tchessalov et al. 2021). Various models have been developed for the critical primary drying phase, based on fundamental mass and heat transfer principles. These models typically yield similar results and are generally founded on one-dimensional unsteady or quasi-steady state heat and mass transfer equations (Pikal et al. 2018; Shivkumar et al. 2019a, b). Key model inputs commonly include vial heat transfer coefficients (accounting for edge effects), mass transfer coefficients (including cake resistance), and equipment capability constraints. While primary drying models are not novel, their adoption as a valuable alternative to scaled experimentation has increased their industrial application for process optimization, scale-up, and technology transfer.

Factors such as ice nucleation temperature, degree of supercooling, and mass transfer resistance may differ between laboratory and production environments. These variations must be addressed during scale-up. Various approaches have been described to account for these differences, including the use of a “GMP factor” to adjust laboratory-derived resistance values when modeling commercial processes. This empirical factor is derived from observed differences in primary drying time between laboratory and manufacturing cycles (Tcheesalov et al. 2021). Another published approach utilized gravimetric and pressure measurements from commercial manufacturing runs to estimate parameters, addressing the limitations of available sensors in GMP environments (Geremia et al. 2022). Also, a coupled 3-D Computational Fluid Dynamics (CFD) and 1-D vial-scale simulation model has been introduced to create a digital twin of lyophilization units, revealing different responses between laboratory-scale and commercial-scale lyophilizers in failure scenarios (Zadravec et al. 2025).

Several case studies from pharmaceutical companies have demonstrated the model’s applications, including optimizing cycle parameters and reducing drying time; scaling up from laboratory to commercial scale with minimal experimental runs; assessing the impact of process deviations on product quality; generating design spaces for different product types, and evaluating new product presentations and vial sizes (Tsinontides et al. 2004; Zhu et al. 2018; Schivkumar et al. 2019a; Tchessalov et al. 2021; Jameel et al. 2021; Vanbillemont et al. 2023). To achieve these outcomes, the process model must be fit for purpose and credible. Risk-based approaches for establishing model credibility have been advanced (FDA 2023; EMA 2024; O’Connor et al. 2024; FDA 2025) and informed by consensus standards such as the American Society of Mechanical Engineers’ V&V 40 – Assessing Credibility of Computational Modeling through Verification and Validation: Application to Medical Device (ASME 2018). In general, these frameworks begin with defining the specific role and scope of the process model used to address the identified question of interest.

Model risk is assessed as a combination of model influence and decision consequence. Credibility assessment activities typically are commensurate with the model risk and tailored to the specific context of use. Industrial case studies of primary drying models published in scientific literature have primarily addressed development-related questions, such as optimizing cycle parameters. The decision consequences of these development decisions, with respect to the product quality of commercially manufactured batches, are generally lower than those of process models used as part of the control strategy in commercial manufacturing.

### Alternate aseptic drying technologies

#### Continuous freeze-drying

In 2015, continuous direct compression became the first of its kind continuous manufacturing (CM) process to be accepted by the FDA for evaluation. This innovative CM-based manufacturing technology offered several advantages: minimizing the need for human interventions; helping improve the assurance of product quality; and enabling manufacturing flexibility and agility (Fisher et al. 2022). Similarly, there has been increased research interest to expand the use of CM for freeze-drying. For example, a concept of CM-based freeze-drying was introduced by using a train of suspended-vials that are subjected to CIN, followed by drying with radiative heat while moving on one track (Capozzi et al. 2019). Another reported example of CM-based freeze-drying is spin freeze-drying (De Meyer et al. 2015; Van Bockstal et al. 2017) where every filled vial is rapidly rotated along its longitudinal axis to create a thin layer of product against the vial wall during freezing, followed by drying with various methods. A commonality between the two aforementioned approaches is that every single vial to be lyophilized is subjected to the same manufacturing conditions. From a manufacturing control standpoint, CM-based freeze-drying has the potential to reduce vial-to-vial heterogeneity in heat and mass transfer generally observed from conventional shelf freeze-drying, thereby improving control of product quality of individual vials. Another manufacturing technology amenable to CM-based freeze-drying is spray freeze-drying, which is designed to produce free-flowing powders in bulk as distinguished from vial freeze-drying (Clénet et al. 2019; Borges et al. 2021). Drug product in liquid is converted to fine droplets by leveraging existing spray processes commonly used for the manufacture of solid oral dosage forms (e.g., spray drying and fluid bed spray granulation), prior to being frozen with the help of a cryogenic medium of choice. Regarding the subsequent drying phase, several research papers have shed light on potential manufacturing process designs that could make spray freeze-drying suitable for CM-based manufacturing (Pisano et al. 2019). In our survey, we did not find any application containing use of this technique to manufacture drug products.

#### Microwave-assisted freeze-drying

Growing interests in microwave-assisted freeze-drying (MAFD) as an alternative to conventional shelf freeze-drying merit attention. Notably, the MAFD is proven to shorten drying time significantly while maintaining product efficacy and stability (Gitter et al. 2018), making it advantageous to the pharmaceutical industry from a manufacturing efficiency perspective. The nature of microwave volumetric heating used in the MAFD is also conducive to making the drying process compatible with frozen products in various types of container closure systems besides vials. However, it is imperative that uniform heating be provided in a controlled manner to minimize drying heterogeneity. To this end, research efforts in elucidating the impact of equipment design and process parameters on heterogeneity in drying behaviors have been made (Bhambhani et al. 2021; Abdelraheem et al. 2022; Alexeenko et al. 2025). In our survey, we did not find any application containing use of this technique to manufacture drug products.

With the advent of emerging technologies in pharmaceutical freeze-drying, improvement of quality control at the individual vial level has become increasingly attainable which minimizes freeze-drying process heterogeneity. In the case of CM-based freeze-drying, care should be taken to make equipment design and system integration suitable for assuring uniformity in freezing as well as heat transfer needed for drying. In addition, the handling of any planned or unplanned manufacturing disruption is important for ensuring consistent quality of the drug product. As demonstrated by a recent study, the use of PAT can help maintain the lyophilization process in a state of control (Leys et al. 2023) where an in-line near-infrared measurement was used to monitor the residual moisture content of every vial in the batch. The same work is also exemplary of active process controls stipulated in the International Council for Harmonisation (ICH) Q13 (ICH 2023) in that the drying phase was controlled by a closed loop system based on in-line measurements of product critical quality attributes. In the case of the MAFD, the challenge of non-uniform microwave heating during drying should be addressed. The selection of critical process parameters and equipment design should consider how much microwave-driven electrical energy is being transferred and dissipated as the heat applied is dependent on equipment as well as drug formulation (e.g., dielectric properties). To spur development and innovation in the area of the MAFD, CDER has recently funded a research project (Fig. 1) aiming to develop a heat and mass transfer model of the MAFD as well as a database of formulation dependent parameters to guide process development.

## Conclusions

Recent trends in process control strategies observed in various types of regulatory submissions and manufacturing inspections have been analyzed for freeze-dried drug products. Our analyses indicated most drug applications surveyed employed certain types of control strategies rather than trial-error approaches to determine the endpoint of drying. The development of emerging technologies in freeze-drying has increased in recent years in the pharmaceutical industry. As with the proven benefit of CM used for solid oral dosage forms, new manufacturing techniques could potentially help the industry build a business case to propel innovation and manufacturing advances in freeze-drying. In the future, active engagement between the industry and the FDA, during early-stage development, would be vital in facilitating successful adoption of emerging technologies in freeze-drying and making use of such highly capable equipment for commercial manufacturing.

## Data Availability

Data cannot be made available due to confidential concerns.

## Abbreviations

ANDA: Abbreviated New Drug Application
AQL: Acceptance Quality Limit
BLA: Biologics License Application
CAPA: Corrective and Preventive Action
CDER: Center for Drug Evaluation and Research
CFD: Computational Fluid Dynamics
CIN: Controlled Ice Nucleation
CM: Continuous Manufacturing
EIR: Establishment Inspection Report
ETP: Emerging Technology Program
FDA: Food and Drug Administration
GMP: Good Manufacturing Practice
HEPA: High Efficiency Particulate Air
ICH: International Council for Harmonisation
ISO: International Organization for Standardization
MAFD: Microwave-Assisted Freeze-Drying
NDA: New Drug Application
PAT: Process Analytical Technology
PPQ: Process Performance Qualification
QbD: Quality by Design
SIP: Sterilization-in-place
TDLAS: Tunable Diode Laser Absorption Spectroscopy
USP: United States Pharmacopeia
